# Dramatic Metabolic Response to Dual ICI With Chemotherapy in Low PD‐L1 NSCLC Case

**DOI:** 10.1002/rcr2.70150

**Published:** 2025-03-16

**Authors:** Toshikazu Kumasa, Akina Nigi, Keisuke Iwamoto, Hidetoshi Itani, Shigeto Kondou

**Affiliations:** ^1^ Department of Respiratory Medicine Japanese Red Cross Ise Hospital Ise Japan

**Keywords:** ipilimumab, low‐PD‐L1expression, lung cancer, nivolumab, PET‐CT

## Abstract

Despite low PD‐L1 (Programmed Death‐ligand 1) expression, this patient exhibited a dramatic positron emission tomography‐computed tomography response to dual immune checkpoint inhibitors with chemotherapy. This case highlights the potential benefits of combination therapy beyond traditional PD‐L1‐based treatment selection.

## Graphical abstract

Metastatic lung cancers are usually difficult to treat. This case highlights the importance of treating with immune checkpoint inhibitors and improving the prognosis of patients with multiple metastases.

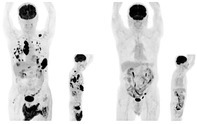

## 1 Clinical Image

**FIGURE 1:**
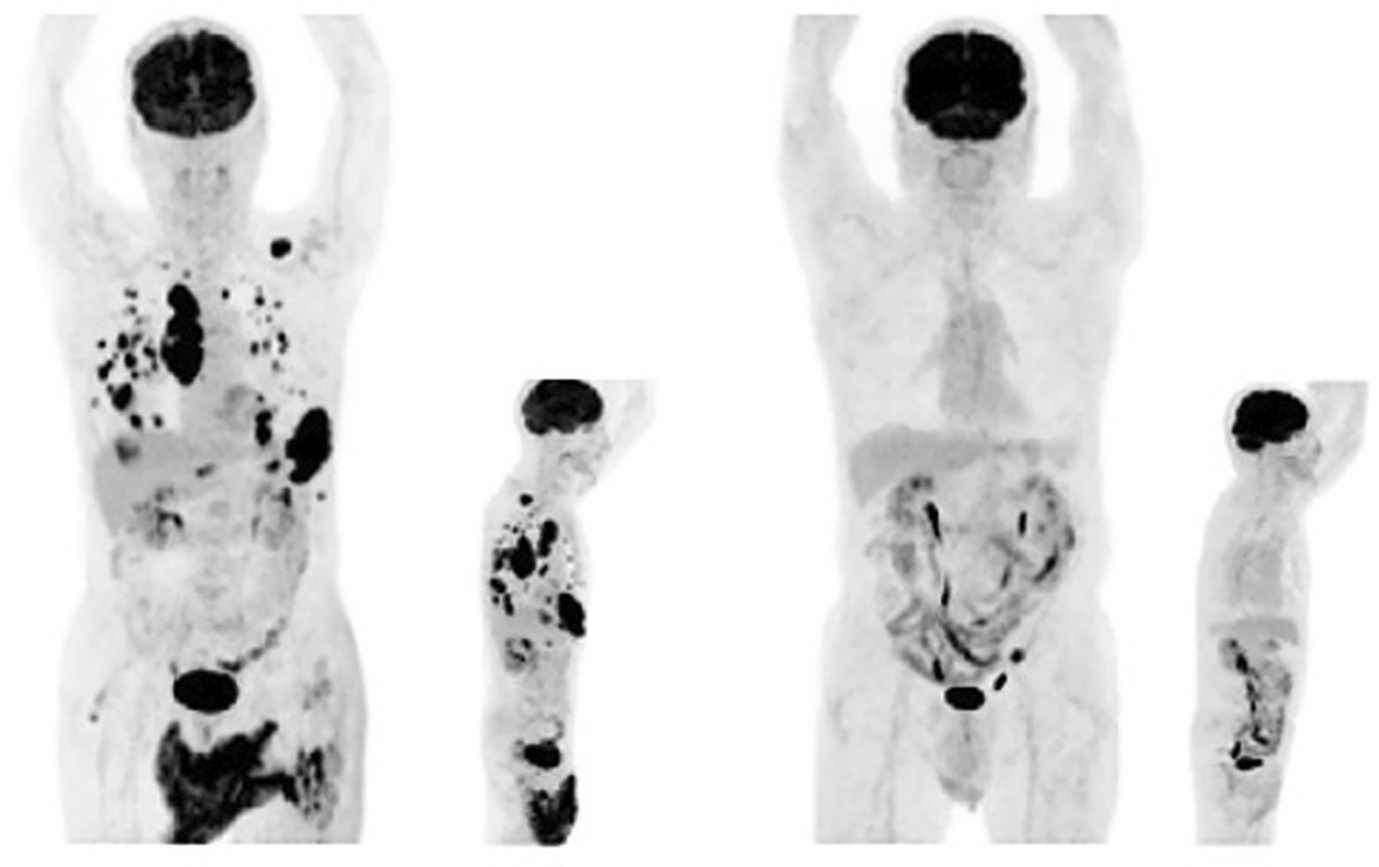
PET‐CT scans before (left) and 14 months after (right) dual ICIs therapy, demonstrating a dramatic reduction in metabolic activity.

We present a case of a 73‐year‐old patient with advanced non‐small cell lung cancer (NSCLC) originating in the right lower lobe, with multiple lymph node metastases, bilateral pulmonary metastases, hepatic metastases, multiple brain metastases, and bone metastases. PD‐L1 (Programmed Death‐Ligand 1) is a cell surface protein that plays a key role in the immune system's regulation of immune response and is an important factor in cancer treatment. The tumour exhibited low PD‐L1 expression (1%). The patient was treated with dual immune checkpoint inhibitors (ICIs) and chemotherapy, receiving two cycles of carboplatin and nab‐paclitaxel, followed by maintenance therapy. Despite the extensive metastatic burden, positron emission tomography‐computed tomography (PET‐CT) imaging performed 14 months after treatment initiation demonstrated a complete metabolic response (Figure 1). This response was sustained for over a year.

The CheckMate 9LA study demonstrated that nivolumab plus ipilimumab with limited chemotherapy improves survival outcomes in metastatic NSCLC, including patients with brain metastases [1]. While extensive metastases, particularly in the brain and liver, are associated with poor prognosis, this case suggests that some patients may achieve prolonged responses with combination ICI therapy. The patient ultimately passed away 25 months after treatment initiation due to unrelated complications. This case underscores the potential for durable responses beyond traditional biomarker‐based selection.

## Author Contributions

All authors reviewed and approved the final manuscript.

## Ethics Statement

The authors declare that appropriate written informed consent was obtained for the publication of this manuscript and accompanying images.

## Conflicts of Interest

The authors declare no conflicts of interest.

## Data Availability

The data that support the findings of this study are available on request from the corresponding author. The data are not publicly available due to privacy or ethical restrictions.
